# *Plasmodium falciparum* Variability and Immune Evasion Proceed from Antigenicity of Consensus Sequences from DBL6ε; Generalization to All DBL from VAR2CSA

**DOI:** 10.1371/journal.pone.0054882

**Published:** 2013-01-25

**Authors:** Philippe Deloron, Jacqueline Milet, Cyril Badaut

**Affiliations:** 1 Institut de Recherche pour le Développement, Mother and Child Faced with Tropical Infections Research Unit, UMR216, Paris, France; 2 PRES Paris Sorbonne Cité, Université Paris Descartes, Paris, France; 3 Centre d'Étude et de Recherche sur le Paludisme Associé à la Grossesse et l'Enfance, Cotonou, Bénin; Centro de Pesquisa Rene Rachou/Fundação Oswaldo Cruz (Fiocruz-Minas), Brazil

## Abstract

We studied all consensus sequences within the four least ‘variable blocks’ (VB) present in the DBL6ε domain of VAR2CSA, the protein involved in the adhesion of infected red blood cells by *Plasmodium falciparum* that causes the Pregnancy-Associated Malaria (PAM). Characterising consensus sequences with respect to recognition of antibodies and percentage of responders among pregnant women living in areas where *P. falciparum* is endemic allows the identification of the most antigenic sequences within each VB. When combining these consensus sequences among four serotypes from VB1 or VB5, the most often recognized ones are expected to induce pan-reactive antibodies recognizing VAR2CSA from all plasmodial strains. These sequences are of main interest in the design of an immunogenic molecule. Using a similar approach than for DBL6ε, we studied the five other DBL and the CIDRpam from VAR2CSA, and again identified VB segments with highly conserved consensus sequences. In addition, we identified consensus sequences in other *var* genes expressed by non-PAM parasites. This finding paves the way for vaccine design against other pathologies caused by *P. falciparum*.

## Introduction

In endemic areas, pregnancy-associated *Plasmodium falciparum* malaria (PAM) causes maternal anaemia, stillbirth and delivery of low birth weight (LBW) babies, the single most important determinant of mortality during the first year of life of African infants. WHO considers malaria in pregnancy as "one of the most important preventable causes of low birth weight deliveries worldwide" and "a major cause of severe maternal anaemia contributing to maternal mortality" [1]. Placental malaria, i.e. the massive infection of the placenta by cytoadhering *P. falciparum* parasites, is a major contributor to PAM. It is more pronounced in primigravidae, where it is associated with a two-fold increased risk to give birth to a LBW baby [2]. PAM is associated with diminished cellular and antibody responses to *P. falciparum* in infants [3]. Infants born to women with PAM are more susceptible to *P. falciparum* infection in the first year of life [4], [5] and is thus a major contributor to infant morbidity and mortality.

*P. falciparum* variable antigens expressed on the infected red blood cell (IRBC) surface are key determinants of the tissue tropism of parasite sequestration. In PAM, sequestration of IRBC in the placenta is mediated by interactions between the variant antigen, VAR2CSA, and chondroitin sulphate A (CSA) displayed on the syncytiotrophoblast surface. The protein is a member of the *Plasmodium falciparum* Erythrocyte Membrane Protein 1 (PfEMP1) family encoded by the *var* multigene repertoire. VAR2CSA, encoded by the *var2csa* gene, is expressed by placental isolates [6]. Evidence is accumulating that the specific immune response against VAR2CSA acquired during the first pregnancies reduces placental sequestration and PAM during subsequent pregnancies. This strongly suggests that the development of a vaccination strategy targeting VAR2CSA is feasible. VAR2CSA is a large protein of 350 kDa [7]. Although the full-length recombinant VAR2CSA protein able to induce adhesion-inhibitory antibodies has been achieved [8], [9], the technological constraints in the production of such a large antigen compromise its use in large-scale vaccination programs. An alternative approach is to produce smaller VAR2CSA fragments presenting epitopes shared among most VAR2CSA variants, i.e. inducing antibodies that inhibit CSA binding of a large panel of VAR2CSA-expressing IRBCs. The constructs explored to date, based on individual recombinant domains, have not been able to induce such broad-reacting inhibitory antibodies. Here, we use a systematic approach to interrogate sequence diversity of VAR2CSA in an attempt to classify the variable regions and to study their distribution within the protein.

The VAR2CSA protein comprises six Duffy Binding Like (DBL) domains, of which three are unclassified, and a Cystein-Rich InterDomain Region (CIDR)pam domain [10]. Compared to other *var* genes, the *var2csa* gene architecture is relatively well conserved between isolates [7], [11]. Nevertheless, the sequence includes both highly conserved and highly variable regions, leading to an identity of 54 to 94% between isolates from different geographic areas [12], [13]. The DBL6ε domain has been identified as the most polymorphic of the six DBL domains of *var2csa*, with a sequence identity ranging from 54 to 61% [14], [15]. Pregnant women who are infected by *P. falciparum* develop antibodies to the DBL6ε domain [16], and this response is parity dependant, being higher in multigravidae than in primigravidae [17]. DBL6ε antibodies bind the surface of RBC infected by PAM isolates and inhibit CSA binding [18]. Using a new approach to compare variable regions in sequences with extensive segmental gene relationship, we demonstrated that the DBL6ε domain of VAR2CSA is composed of seven variable blocks (VB) with limited polymorphism. Accordingly, each VB is composed of a limited number of consensus types and the domain is formed by the combinatorial association of these consensus sequences. Peptide-based ELISA demonstrated that variable blocks with at least 85% average percent pairwise identity (APPI) were equally well recognized by antibody and can thus be grouped into a single consensus serotype. Here, we studied all consensus sequences from the four least variable VB of DBL6ε to determine the most pertinent sequences to be included in a vaccine candidate.

## Materials and Methods

### Studied cohort

The present study was conducted in Thiadiaye Hospital (Thiadiaye is a town situated 130 km east of Dakar, the political capital of Senegal) and in 2 corresponding health centers in the neighbouring villages of Fissel and Sandiara. Malaria there is seasonally transmitted, during the rains from September to December, with an estimate of 10 infected bites/individual/year]. Selected women (n = 80) were actively followed for the duration of their pregnancy, over which they received chloroquine prophylaxis [16]. Of these selected women, 39 (49%) were infected at least once with *P. falciparum* and 41 (51%) were not infected. Among the infected women, six were still infected at delivery, while two others were infected at delivery but were not found to be infected throughout their pregnancy. Their mean age was 24 years. Twenty-eight were primigravidae, 21 secondigravidae, and 31 multigravidae (>2). Eight men and six women living in non-endemic area were used as a negative control. Collected plasma and purified antibodies have been described in [19]. Plasma samples of women were collected at delivery. The non-infected group was composed of 16 primigravidae, 12 secondigravidae, and 15 multigravidae (3–9). The infected group comprised 12 primigravidae, 9 secondigravidae and 16 multigravidae (3–9). The National Ethics Committee of the Senegalese Health Ministry and the Science and Health Faculty Ethics Committee in Benin approved these studies. Written informed consent for data and blood collection and storage was obtained from all subjects before enrolment in the study.

### Pregnant women antibody recognition of DBL6ε VB

ELISA assays were carried out as described in Badaut *et al*. [19]. IgG were purified from all women and control plasma samples, and their reactivity against 15 peptides carrying the three or four consensus sequences from the four less variable blocks (VB1, VB2, VB4 and VB5) present within the DBL6ε domain were measured. All peptides were provided by Genepep and Millegen corporations, and were designed as follows: Biotin-Ahx-GG-peptide-COOH. IgG were purified by incubating 30 µL of plasma on protein G columns to avoid saturation, and to determine the quantity of IgG present in the plasma sample. Ninety-six well plates coated with streptavidin (NUNC) were incubated with 200 µL of blocking buffer (0.5% milk, 0.04% Tween 20 in PBS) at 4°C overnight and washed three times (PBS, 0.04% Tween 20). A volume of 100 µL containing 3.10^−11^ moles of each peptide diluted in blocking buffer was incubated for one hour at room temperature followed by three washing steps. One hundred µL of IgG from pregnant women or control individuals at a concentration of 0.0205 g.L^−1^, diluted in blocking buffer were incubated for 60 min at room temperature. After three washing steps, horseradish peroxidase-conjugated anti–human IgG (1∶3500) was incubated for one hour at room temperature. Tetramethylbenzidine was added and the reaction was stopped after 15 minutes by adding 50 µL of 0.25 M H_2_SO_4_. The optical density (OD) was read at 450 nm. To determine the percentage of responders, for each peptide positive threshold value was calculated by addition of 2 standard deviations to the mean of control individuals’ IgG recognition to each peptide.

### Statistical methods

We compared the total number of women recognizing VB, between the groups of women infected or not by *P. falciparum* during their pregnancy using a Mann–Whitney *U* test. Then the effect of both factors, infection during pregnancy and parity (coded primigravidae, secondigravidae, and multigravidae) were evaluated simultaneously using a negative binomial regression with adjustment for age. An interaction between infection during pregnancy and parity was included in the model to test whether infection during pregnancy effect can depend on parity group. For each VB, we compared the level of the antibody response between infected and non-infected women using the Mann–Whitney *U* test. Analyses were done using the R software package (version 2.10.1, http://www.r-project.org).

### Sequence analysis - alignment

Sequences were recovered from databanks (for accession number, see left column on figures). First, five sequences from each DBL were aligned altogether to identify highly conserved blocks that, combined with the structure model analysis, allowed identifying variable blocks. Secondly, all DBLs were aligned independently. Twenty-nine DBL1X, 65 DBL2X, 14 CIDRpam, 89 DBL3X, 14 DBL4ε, 39 DBL5ε, and 29 DBL6ε [19] sequences were identified in the databanks. Alignment was done with the Geneious software, using the plug-in Muscle program. Amino acids were coloured according to their similarity.

### Epitope B prediction

Linear B-cell epitopes were predicted using ElliPro website (http://tools.immuneepitope.org/tools/ElliPro/iedb_input) with default settings, and the 3BQK structure of DBL3X domain [20]. VB linear epitopes were coloured on the structure to compare their location.

## Results

### Immunogenicity of consensus sequences found on the surface of DBL6ε domain, within the four least variable VB

**Figure 1:**
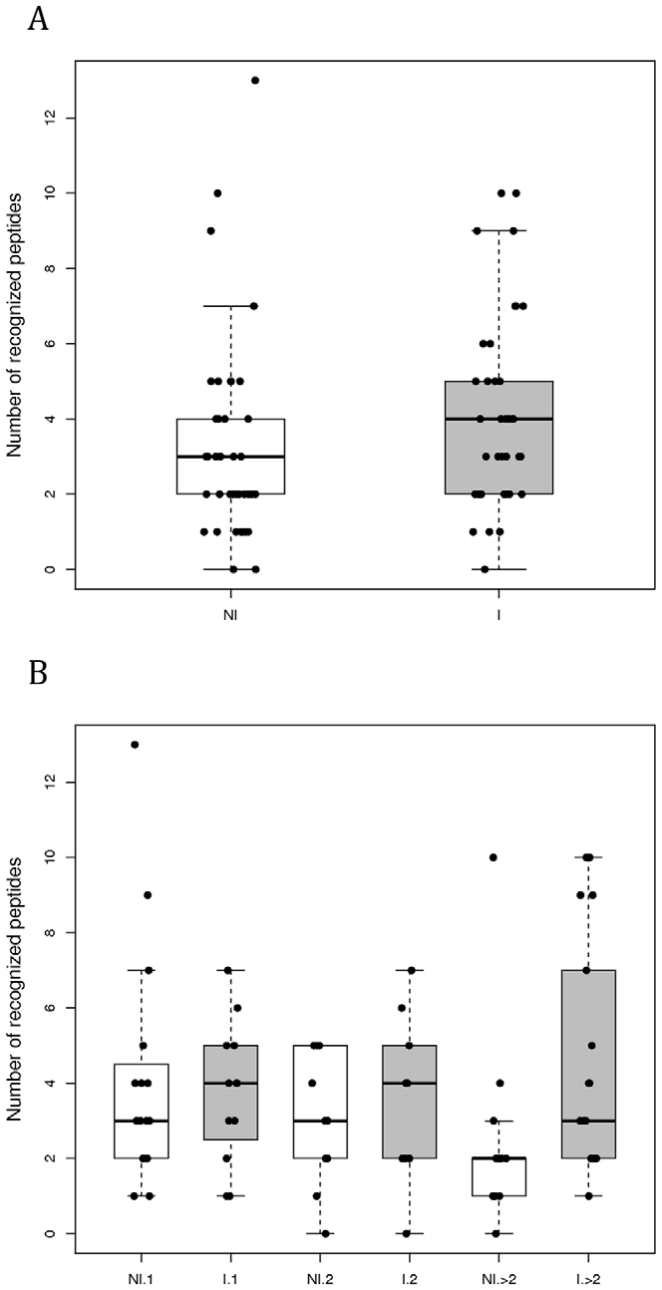
Number of consensus sequences recognized by purified IgGs. (**A**) From the plasma of pregnant women having been infected (n = 39) or not (n = 41) by *P. falciparum* during their pregnancy; (**B**) according to parity. The top, bottom, and middle lines of the boxes correspond to the 75^th^ percentile, 25^th^ percentile, and 50^th^ percentile (median), respectively. The whiskers extend from the 10^th^ percentile and top 90^th^ percentile.

The variable blocks (VB) and their associated consensus sequences have been characterised in [19]. Briefly, local alignment has been performed considering each VB. All sequences with at least 85% of pairwise identity have been considered in the same clade and associated with one consensus sequence. We compared the number of VB peptide sequences recognized by protein G purified IgG from the plasma of women infected by *P. falciparum* (n = 39, mean age = 24) or not infected (n = 41, mean age = 25) during their pregnancy (Figure 1A). The group of infected women included the women infected during their pregnancy (n = 37) or at delivery (n = 2). Four variant consensus sequences of VB1, 2 and 5 were studied alongside three variant consensus sequences of VB4 (for sequences see Table 1). Infected women tended to recognize a higher number of sequences (median = 4; interquartile = 2–6) than non-infected women (median = 3; interquartile = 1–4) (p = 0.067). In multigravidae group, we observed a higher difference between non-infected and infected women (Figure 1B), non-infected multigravidae women recognizing fewer sequences than primigravidae and secondigravidae. But this difference was not significant in multivariate analyses (p = 0.088). Negative binomial regression does not show any effect of age or gravidity on the number of recognized sequences.

**Table 1:** Synthesized peptide sequences.

VB	Sequence
VB1-1	Biot-AhxGGKNLYSRMQHNIDTIWTDLLVKNSSDINK
VB1-2	Biot-AhxGGKNINVNMKKNNDNIWTDLLVKNSSDINK
VB1-3	Biot-AhxGGKNINVNKRHKNDDTFVTDNFVKKSWEISN
VB1-4	Biot-AhxGGKNIHDRMKKNNGNFVTDNFVKNSWEISN
VB2-1	Biot-AhxGGNIKDTQICQYKRDPKL
VB2-2	Biot-AhxGGYIDPSKICEYKKNPKL
VB2-3	Biot-AhxGGKIDESDICEYKKDPKL
VB2-4	Biot-AhxGGNIEKSDICKYKKNPKL
VB4-1	Biot-AhxGGSPTSKYIEQIFKGTEYSGIDSET
VB4-2	Biot-AhxGGKNSSDKIGKILGDTDGQNEKRKK
VB4-3	Biot-AhxGGNNSSDNIGKILGGDGDRKNEKRKA
VB5-1	Biot-AhxGGKHAYGNISHDDKKKLDIPNNDNEH
VB5-2	Biot-AhxGGQKGKNNTHSEKLDEAWCDLPMEGDIP
VB5-3	Biot-AhxGGKQAGGYKETVEKLSEKKQKCTFPDIESVP
VB5-4	Biot-AhxGGKQAGGDTKTNENCRFPDTDGVP

Peptides were designed to represent all consensus sequences found within each of VB1, VB2, VB4, and VB5 sequences. An arm of an ε-aminohexanoic acid (Ahx) and two glycin residues was added to each sequence.

IgG of 42% non-infected and 57% infected women (Table 2) recognized at least one consensus sequence among the four sequences within VB1. IgG from 58% and 70% of non-infected and infected women, respectively, recognized at least one consensus sequence from the group formed with VB1 plus VB5. IgG from 70% and 86% of non-infected and infected women, respectively, recognised at least one consensus sequence among all VB. Since consensus sequences from VB are present in parasites from all endemic areas [14], [19], our results suggest that a vaccine constituted by these peptides from these four VB could induce antibodies against essentially all *P. falciparum* isolates involved in pregnancy-associated malaria.

**Table 2:** Percentage of responders against group of sequences formed within VB.

	VB1	VB2	VB4	VB5	VB1+VB5	all
Non-infected (%)	42	9	23	35	58	70
Infected (%)	57	16	29	54	70	86

Percentage of responders (with a OD higher than mean +2 standard deviations of European women response) that recognized group of consensus sequences within each VB, within both VB1 and VB5, or within all VB shows that almost all women have IgG reacting with at least one of the 15 studied consensus sequences, with 85% APPI.

**Figure 2:**
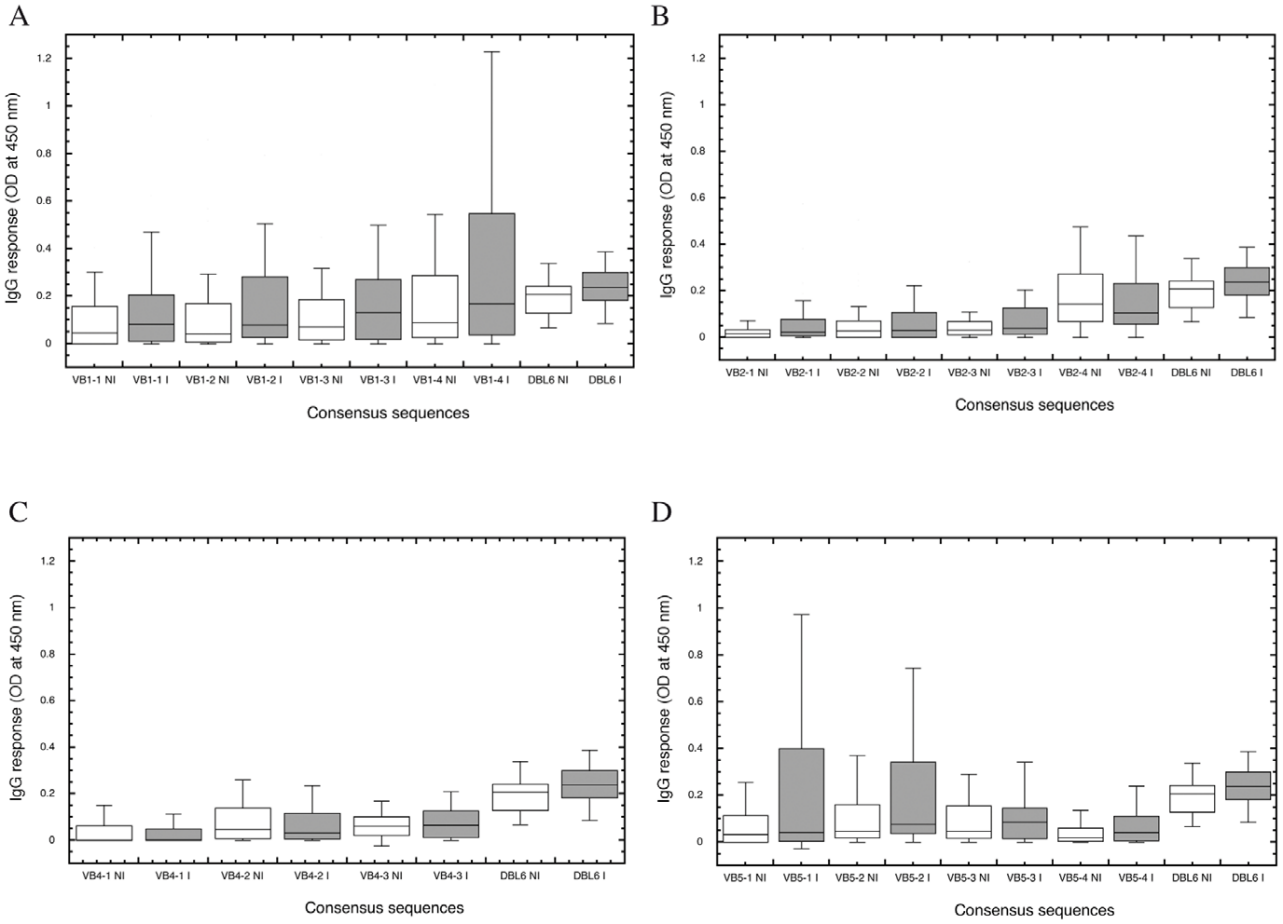
Intensity of the recognition of consensus sequences. Consensus sequences determined for each VB were tested by ELISA for their recognition by purified IgGs of infected (I.) or non-infected (NI.) pregnant women. For VB1 (**A**), VB2 (**B**), VB4 (**C**), and VB5 (**D**), the top, bottom, and middle lines of the boxes correspond to the 75^th^ percentile, 25^th^ percentile, and 50^th^ percentile (median), respectively. The whiskers extend from the 10^th^ percentile and top 90^th^ percentile. The level of response to DBL6ε–CYK48 [19] of infected and non-infected women was added on the right part of each graph to compare the VB response intensity.

The IgG response against the 15 consensus VB peptides was compared between infected and non-infected women (Figure 2). Purified IgG from infected women recognized the consensus sequences from VB1 to a higher level than IgG from non-infected women (Figure 2A). This difference was observed for all VB1 consensus sequences, being of borderline significant for the VB1-2 consensus sequence (p = 0.051). The recognition of the consensus sequences of VB2, except VB2-4, was also higher with IgG purified from the plasma of infected women (Figure 2B). Within the VB5 group, VB5-1 and VB5-2 (Figure 2D) recognition tended to be higher with IgG from infected women than from non-infected women (p = 0.067). Conversely, VB4 consensus sequences were equally recognized by the two groups (Figure 2C).

**Figure 3:**
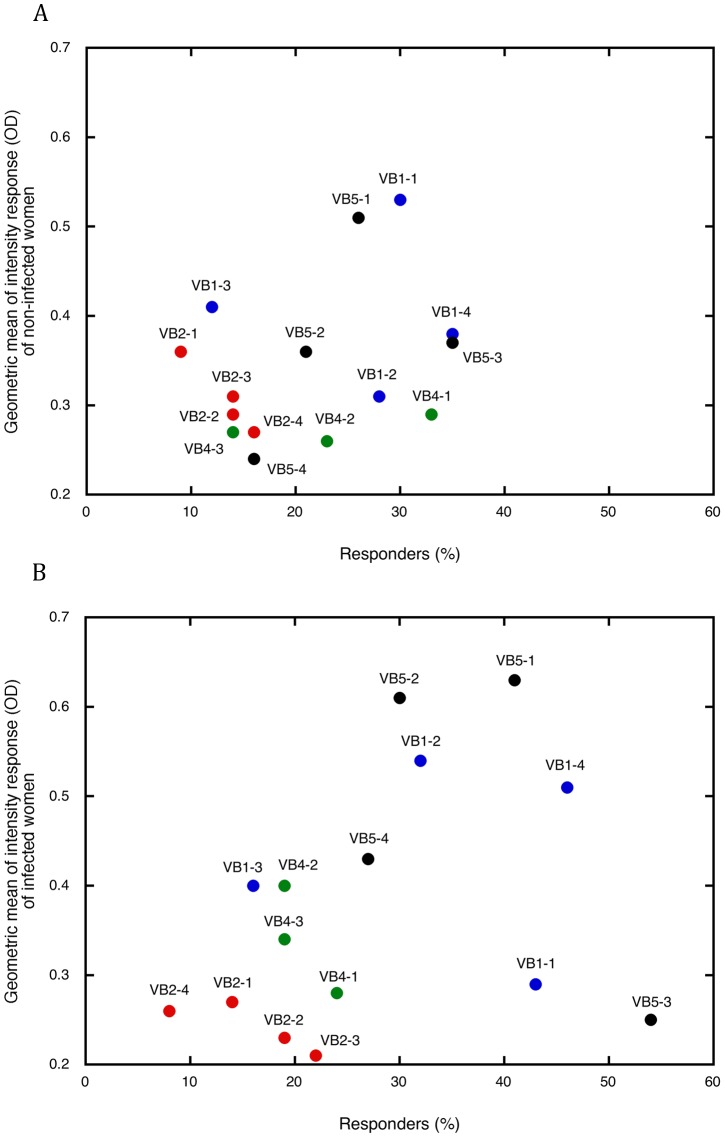
Characterization of the recognition of consensus sequences from 4 VB from DBL6ε by purified IgGs from the plasma of pregnant women. ELISA reactivity of VB1 (blue), VB2 (red), VB4 (green), and VB5 (black) is plotted on a graph. Along X, the percentage of responders, and along Y, the geometric mean of the antibody response of responders within non-infected (**A**) or infected women during pregnancy (**B**). A positive response is defined as an IgG response greater than the average of the values of its group +2 standard deviations of European women response. Repartitions of dots differ in infected and non-infected women: dots of non-infected women being grouped while those of infected women are sparse.

To better characterize the response to each variant consensus sequence of VB1, VB2, VB4, and VB5, we correlated the percentage of responders to a given peptide with the mean level of IgG response to that peptide (Figure 3). Both non-infected (Figure 3A) and infected women (Figure 3B) responded to several VB sequences, which we compared by examining the clustering of points on these graphs. When women were not infected during their pregnancy, the points were quite clustered and both geometric means of the responses and percentages of responders were low. Within the infected women group, the points were more dispersed; moreover, the geometric means and the percentages of responders were higher than in non-infected women. The strong responses to the VB1 peptides highlights the importance of these consensus sequences, especially VB1-2 and VB1-4. Within the infected group, IgG from a high percentage of women showed a significant response to the peptides (geometric mean = 0.54 and 0.51 for VB1-2 and VB1-4, respectively). The antibody response to antigens carrying these VB reacted with the corresponding sequences, and this response should be boosted in case of *P. falciparum* infection during pregnancy.

VB2 consensus sequences were poorly recognised, with a low level signal and a low number of responders. Only few IgG recognized the VB4 peptides. Seroprevalence to VB4-1 was lower in infected compared to uninfected women (32% vs. 23%). The levels of response to VB4-2 and to VB4-3 were higher in infected than in non-infected women, but again the number of responders was low. The VB5-1 and VB5-2 were recognized by 41 and 30% of infected women IgG with a mean level of 0.63 and 0.61, indicating these sequences are major antibody targets. Two consensus sequences, VB1-1 and VB5-3, were less well recognized in non-infected women.

### Variable blocks and consensus sequences are also present in the other domains of VAR2CSA

Studies of DBL sequences led to 10 semi-conserved homology blocks characterisation [10], [14], [21], and pointed out the presence of consensus sequences specific of the DBL classes and subclasses (DBL1pam, DBL2pam, DBL3pam, DBLεpam4, DBLεpam5, DBLpam6 and CIDRpam). Hyper-variable blocks were delimited summarizing the sequence in a WebLogo. Here, we followed these findings, and aligned all DBL from VAR2CSA, providing other blocks information, more precise in length and sequence.

**Figure 4:**
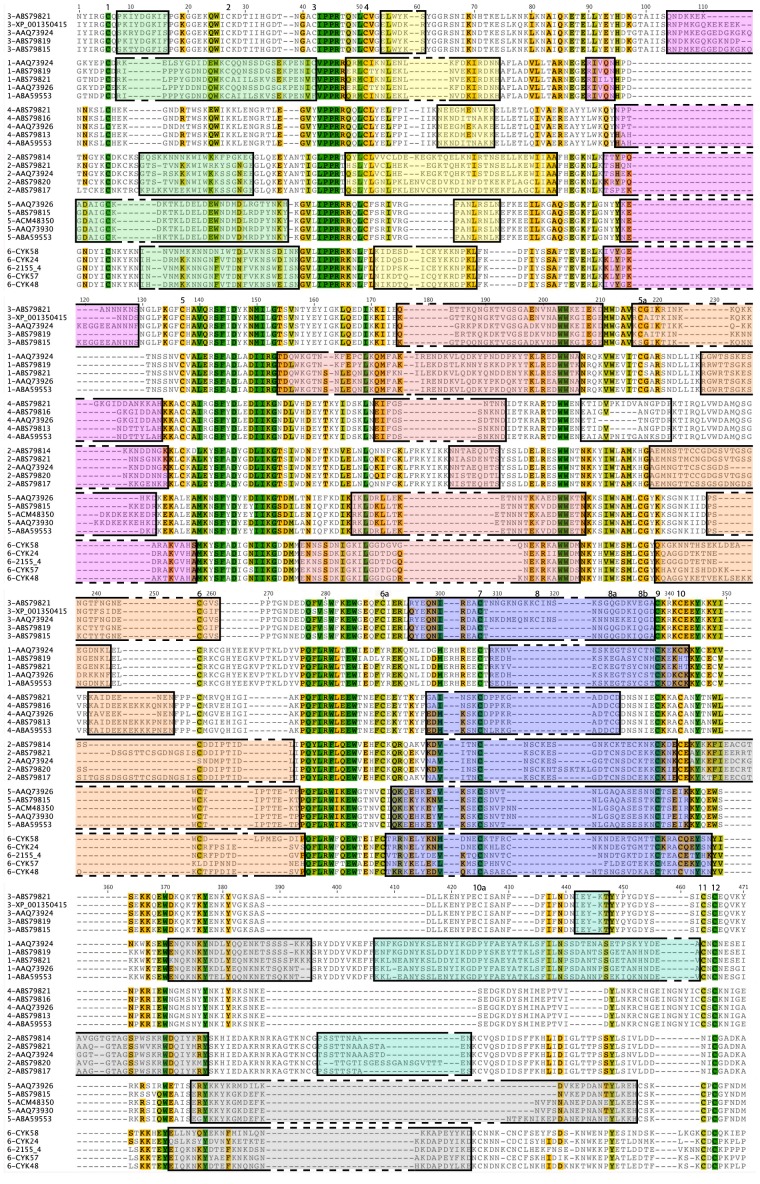
VB localisation and characterisation on each DBL from VAR2CSA. Five DBL1X, DBL2X, DBL3X, DBL4ε, DBL5ε, and DBL6ε from VAR2CSA were chosen from data banks to have the least similar sequence within their VB and were aligned to determine and compare the localization and the length of the VB. Amino acids were coloured following their similarity: green: 100% similar; khaki: 80 to 100% similar; orange: 60 to 80% similar; gray: less than 60% similar. VB1, VB2, VB3, VB4, VB4 extension, VB5, VB6, VB7, and VB7 extension were coloured in green light, yellow, pink, red light, orange, uncoloured, purple, gray and green. Each sequence is labelled on the left by the DBL number and ID in data banks. Order of DBL in this alignment is related to their sequence identity.

**Figure 5:**
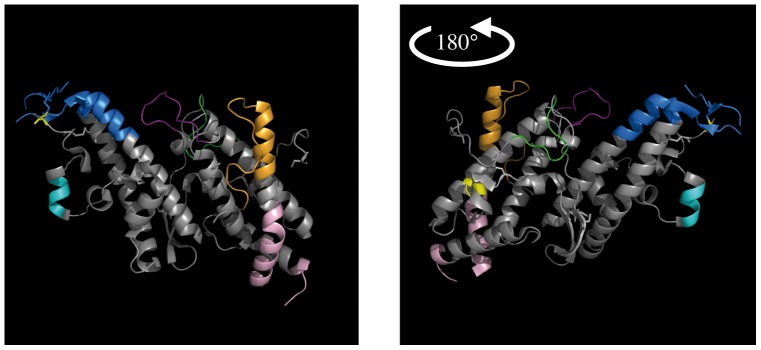
VB localisation on the DBL3X structure. VB1, VB2, VB3, VB4, VB5, VB6, and VB7 are coloured in green, yellow, dark purple, pink, orange, red, blue and turquoise blue on the DBL6ε structure. Both figures represent the protein with a 180° rotation.

To determine the presence and the characteristics of VB within the various DBL and CIDRpam domains from VAR2CSA, we performed a global alignment (Figure 4), as described for the DBL6ε [19] and DBL3X domains [22], and the full-length VAR2CSA protein [14]. Our principal goal was to find the largest non-degenerated sequences (constant blocks or homology blocks) that allow, in a second step, to delimitate all variable blocks. A first alignment permitted to delimit constant blocks, as already described [10], [14], [21], but without defining non-degenerated sequences. Their sequences are DBL-class and subclass specific but, for example, the strict sequence of the constant block located between VB1 and VB2, flanking the PPR motif, is highly specific for the DBL localisation within the VAR2CSA (VPPRR for DBL1X, EYANTIGLPPRT for DBL2X, TNGACIPPRTQNLCVG for DBL3X, LEGVYVPPRRQQLCLY for DBL4ε, KGVLIPPRRRQLCFSR for DBL5ε, and GVLIPPRRKNLFL for DBL6ε), as already described as non-degenerated sequences [10]. This study has already discriminated the DBL location by reclassifying DBL from VAR2CSA as DBLpam1, DBLpam2, DBLpam3, DBLpam4, DBLpam5 and DBLpam6 (also referred to as DBLε10). We used these constant blocks (without any degenerated sequences) to define the precise limit of the VB between them. We made a local alignment, and group each sequence of these variable blocks within various clade. This step was very closed to the ‘type expended alignment’ described in [14]. We compared them in terms of number of amino acids and length variability (supplemental Figures S1 for DBL1X, S2 for DBL2X, S3 for CIDRpam, S4 for DBL3X, S5 for DBL4ε, S6 for DBL5ε, and Figure 2 in [19]). This observation was partially done [14], but local alignment of VB permits to define precisely consensus sequences. The DBL3X structure (Figure 5) was used as a template to localise the VB sequences within the secondary structure motifs (β-sheet, α-helix or coil) and to permit the comparison between the VB of the DBL3X and DBL6ε structures. We had previously observed that the consensus sequences of the DBL6ε domain were VB-specific. Here, we confirm that a given consensus sequence is specific to the domain type: one sequence cannot belong to more than one determined VB and one specific DBL within the VAR2CSA protein; each sequence is a signature of the place of a given DBL within the VAR2CSA protein.

VB1, located in the N-terminal region (amino acids 1–42 highlighted in green, Figure 4), is present in all DBL domains except DBL4ε. This VB is located in a loop on the surface of sub-domain 1 (green coloured amino acids of DBL3X, Figure 5. In DBL1X, DBL3X and DBL5ε, VB1 is constant in length, with 29, 9 or 30 amino acids, respectively, and displays many conserved amino acids; however, it is variable in DBL2X and DBL6ε with 19 to 20 and 25 to 27 amino acids respectively. In DBL1X, DBL2X and DBL6ε, the VB1 sequence includes a highly variable part and a conserved region. Sequence alignment shows two different groups (amino-terminal and carboxy-terminal) represented by two half-consensus sequences (for example, VB1 of DBL1X has two parts: the N-terminal one, WQC, and the C-terminal one, see supplemental Figure S1). The highly conserved sequence L-V-I/PPR/R-T (around amino acid 44 in Figure 4) is shared by all DBL domains, and separates VB1 and VB2.

VB2 (amino acids 48–84 highlighted in yellow on Figures 4 and 5) observed on DBL6ε and DBL3X structures is localized in a loop connecting the amino terminus part of the α-helix 1 found in the DBL6ε structure, and makes the link between sub-domain 1 and sub-domain 2. Its length is constant in the DBL1X, DBL3X, DBL4ε, DBL5ε, and DBL6ε (with respectively 20, 6, 10, 8, and 13 amino acids), but is variable in DBL2X (from 35 to 38 amino acids). The distribution of variable residues in VB2 is also DBL dependent: in DBL2X and DBL4ε, the variability is more dispersed along the sequence, while in DBL1X, DBL3X, DBL5ε, and DBL6ε, only few amino acids are variable. The sequence between VB2 and VB3 (around amino acid 85) is not conserved among the six DBL domains (see Figure 4, no conserved amino acid track (dark green) is observed).

VB3 (amino acids 90–140 highlighted in purple, Figure 4) is present in a loop (DBL3X, in purple Figure 5) and the two parts of its flanking α-helices 1 and 2. The VB3 length is variable in the DBL2X, DBL3X, DBL4ε, and DBL5ε (with respectively from 9 to 21, 13 to 37, 10 to 14, and 5 to 13 amino acids), but is constant in DBL1X and DBL6ε (with respectively 5 and 13 amino acids). The variability of VB3 is dispersed over the whole sequence for DBL2X, DBL3X, DBL4ε, DBL5ε and DBL6ε, but is more clustered in DBL1X. The highly conserved sequence SFXD (around amino acids 139/153) between VB3 and VB4 is shared by all DBL domains.

VB4 secondary structure (amino acids 155–210 highlighted in pink, Figure 4), as observed in structures of DBL, straddles the α-helix 3, the connecting loop, and a part of the α-helix 4 (amino acids coloured in pink on DBL3X, Figure 5). The VB4 lengths of DBL1X, DBL2X, DBL5ε, and DBL6ε vary, with respectively from 49 to 53, 26 to 30, 25 to 26, and 24 to 25 amino acids. The DBL2X has 9 amino acids. Variability of most VB4 is distributed over the entire sequence. VB4 from DBL4ε and DBL5ε have conserved amino acids in their sequences (for DBL5ε: KLD, KETNN and EDWW see Figure S6). In DBL1X, DBL2X, DBL3X, DBL5ε, and DBL6ε, the tryptophan in position 212, localized in the middle of the α-helix, delimits either the carboxy terminal part of VB4 and the amino terminal part of VB5, or the extension of VB4 for DBL4ε (Figure S5). Indeed, VB4 of DBL4ε has an extension localized in the middle of this helix 4. This VB4 extension (amino acids 207 to 222 coloured in white, Figure 4) is variable in length (from 9 to 16 amino acids). Variability is distributed over the sequence but 3 consensus sequences are observed.

The VB5 (amino acids 216–286 highlighted in orange, Figure 4) is localized in the carboxy terminal part of the α-helix 4 and in the amino terminal of the loop connecting the two sub-domains 2 and 3. The length of this VB is highly variable for DBL2X (23 to 42) and repetition of the sequence TTCSSGSGS observed up to 3 times (Figure S2) makes this VB highly variable. The VB5 length varies for DBL3X, DBL4ε, and DBL6ε (from 21 to 25, 7 to 14, and 22 to 29 amino acids respectively), but only few consensus sequences are counted for each of them. The VB5 sequence of DBL4ε contains numerous lysine and glutamate residues. VB5 of DBL1X and DBL5ε have 15 and 12 amino acids, respectively, most being conserved. The sequence delimiting VB5 and VB6 is highly conserved (dark green amino acids positioned around 280, Figure 4) among the different DBL domains.

The VB6 is present in all DBL domains (amino acids 291–347 highlighted in blue, Figure 4) and is localized in the carboxy terminal part of the α-helix 5, the connecting loop, and the amino terminal part of α-helix 6 of the sub-domain 3 (dark blue amino acids in Figure 5). Its length is variable for DBL2X, DBL3X, DBL5ε, and DBL6ε with respectively 20 to 38, 23 to 34, 34 to 36, and 38 to 40 amino acids. Those present in the α-helices are most often conserved, while amino acids localized in the loop are all variable (variability due to few consensus sequences, as reported [19]). VB6 from DBL1X and DBL4ε have 21 and 17 amino acids length, and their variability is low. The sequence between VB6 and VB7 is not highly conserved among all DBL, but the tyrosine residue 345 is always present.

With regard to the structure of DBL6ε, VB7 is localized in the C-terminal region of α-helix 6, a loop, and a small α-helix (Figure 2 in [19]). Our VB references are those of the DBL6ε domain, following sequential (amino acids 344–452 highlighted in grey, Figure 4) and structural alignment. VB7 of DBL2X positioned close to VB6, separated by 2 conserved amino acids (CE). Moreover, the region of DBL3X and DBL4ε aligning with VB7 in other DBL domains is not variable; this VB is therefore absent in these two domains. The length of this block is always variable with 20 to 22, 32 to 39, 24 to 36, and 25 to 27 for DBL1X, DBL2X, DBL5ε, and DBL6ε, respectively. Variability of these sequences is high, as in DBL6ε, but is not homogenous; a part of VB7 is composed of amino acids highly conserved (coloured in red in Figures S1, S2, and S6) and the other composed of variable amino acids (coloured in black). DBL1X and DBL2X have an extension to VB7 (amino acids 396–463 highlighted in light green, Figure 4) of 47 to 52 and 8 to 20 amino acids, respectively. The DBL3X domain has no VB7 (Figure 5), but a variable segment is present on the C-terminal region (at amino acids 442) that can be considered as a VB7 extension, with a length of 5 residues.

### Colocalisation of VB and linear B-cell epitopes on the DBL3X structure

**Figure 6:**
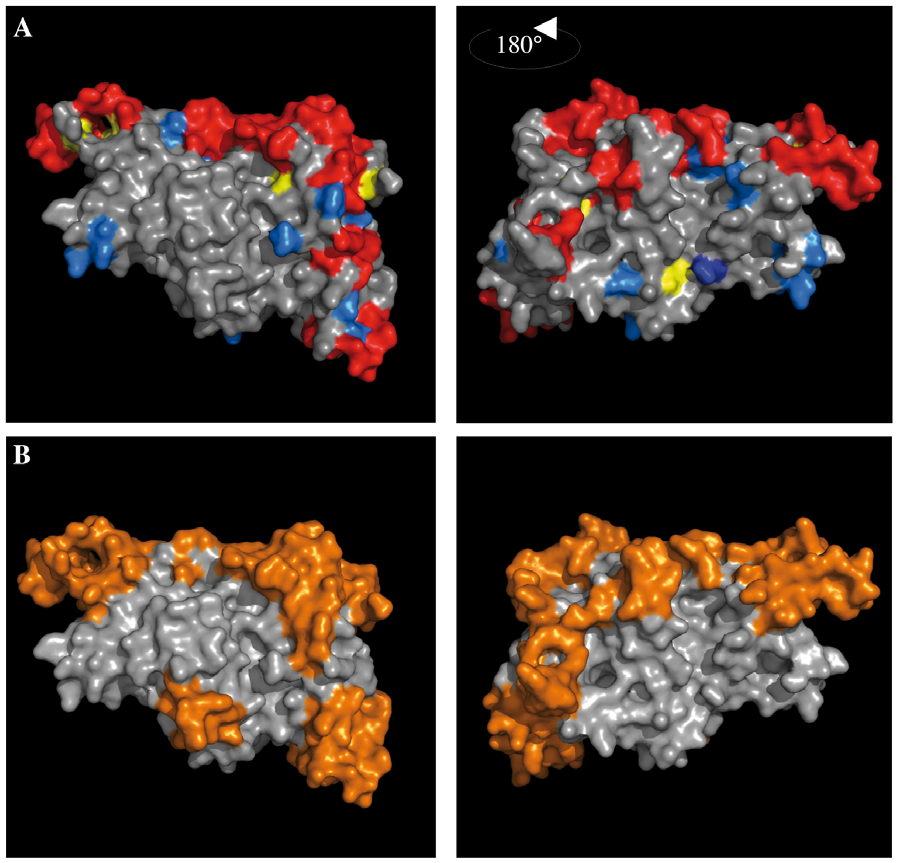
VB and predicted linear and conformational B epitope localisation on the DBL3X structure. On the DBL3X structure of DBL6ε (PDB: 3BQK) were localized: **A**) variable blocks coloured in red, isolated mutated amino acids in bleu, and in **B**) the predicted linear B epitopes are coloured in orange.

Variable blocks of the DBL3X domain are surface exposed (coloured red in Figure 6) and are clustered along an edge, which highlights an area (in gray) that may not be accessible to antibodies. Some single variable amino acids (in blue) are scattered on the surface of the domain. The predicted linear B-cell epitopes are mainly localized to the VB. Foremost, this prediction reinforces our hypothesis (an assumption already made by [14]) that variable blocks are exposed to the immune system and could constitute efficient B-cell epitopes. Moreover, regions of little or no variability (e.g., the absence of variable region corresponding to VB1 in DBL4ε) are likely not implicated in immune escape.

## Discussion

Conflicting results have been observed in experiments aimed at determining the recognition of various VAR2CSA domains by antibodies from infected pregnant women, in particular with DBL6ε [16], [17], [18], [23], [24], [25]; each laboratory studied a different DBL domain cloned from different strains [16], [22], [24], [26], [27], [28], [29], [30], [31]. A recent study on a small number of allelic forms of each VAR2CSA DBL [32] showed large differences in IgG recognition between a given domain from various strains; for example, between the DBL6ε domains of 7G8 and FCR3 (5% and 42%, respectively). IgG recognition arises from the sum of several epitopes on an antigen, and one DBL should carries some epitopes, few of them being surface exposed. These many possible sequences give rise to the high variability of serotypes found in malaria endemic areas. In our previous work [19], we described the low variability of the DBL6ε domain, focusing on variable blocks found on the surface of this domain from one isolate. Here, we extend our findings to all consensus sequences to determine the optimal sequences from the four less variable VB for vaccine development. We studied recognition of consensus sequence of DBL6ε by IgG from pregnant women to understand the potential antibody-driven pressure on diversification. Antibody recognition of these consensus sequences tended to be better in pregnant women having been infected with *P. falciparum* during pregnancy. Although no statistical significance was achieved, the number of recognized sequences or the IgG response against each VB peptide (some being borderline significant) were always higher in women infected during pregnancy. This suggests that the infection induced antibody targeted to these sequences of the DBL6ε domain. Some consensus sequences are better recognized than others. Such differences could explain the conflicting results observed in previous studies as they were conducted with constructs from a single isolate [16], [17], [18], [24], studying only one sequence within each VB. Conversely, multigravidae appear to not recognize more sequences than primigravidae, and response to peptides and parity were not correlated. Certainly, such a correlation would have been observed with the antibody response to crude parasite antigen or with a recombinant protein. However, we did not follow antibody reactivity against full antigens of the pathogen, but only against four epitopes, and more precisely against three or four “serotypes” of each. The probability to be infected with exactly the same sequence in two pregnancies is certainly low, and the antibodies we measured are likely induced by a single infection, and therefore, there is no parity-related increase in the antibody level.

We determined 15 consensus sequences from the four less variable blocks (VB1, VB2, VB4, and VB5 with a variability factor of 0.40, 0.24, 0.34, and 0.37, respectively [19]). This variability factor considers, following a local alignment, the similarity between sequences from each clade. We found that VB1 and VB5 are the best-recognised blocks in the DBL6ε domain. With regard to the percentage of responders, the antibody level and the difference in antibody response between infected and non-infected pregnant women to VB1 and VB5, certain consensus sequences (VB1-4, VB5-1, and VB5-2) are promising for vaccine design if included with a T-cell epitope [33], [34], [35], [36], as a linear polypeptide [37] or a Multiple Antigenic Peptide (MAP) [35], [38]. Because of their length, physico-chemical characteristics, water solubility, stability and large-scale synthesizability, peptides are highly adapted to this end. Their surface localisation in the isolated DBL structure and their variability [19] suggest that they are solvent accessible on the surface of the PfEMP1 protein. Their variability (represented by a small number of consensus sequences) confirms they are major IgG targets in the full-length extra-cellular region of VAR2CSA. The lower variability of VB2 and VB4 may be correlated with their poor recognition by immune IgG. Moreover, their structure as isolated peptides may also be very different from that in the protein.

So far, no study has investigated the protective role of antibodies targeting these VB. We have not demonstrated that these VB are the targets of adhesion-blocking IgG but this is suggested by the high level of polymorphism of these sequences. However, selected DBL6ε serotypes have been shown to be the target of adhesion-blocking antibodies [18]. The demonstration that VB-affinity purified antibodies recognize the protein displayed on the surface of the IRBC is needed to validate the hypothesis of the surface accessibility of the VB. Finally, a single protein that has one allele of each VB should be also able to induce antibody directed to a large number of *P. falciparum* strains. The four consensus sequences of VB1 and the four of VB5 should be sufficient to form the basis of a pan-reactive vaccine since antisera raised against the DBL6ε domain display adhesion-blocking activity [18].

To better understand the polymorphism and the distribution of the variable blocks in the VAR2CSA sequence, we performed a global alignment of all available sequences of all DBL from VAR2CSA, as we previously did for DBL6ε [19]. Our results corroborate those of Bockhorst *et al* [10], [14], [21] and Hommel *et al* [22]. We first determined the highly conserved sequences common to the VAR2CSA DBL scaffolds (Figure 5). Between these conserved blocks, we were able to identify variable ones. Most of them possessed consensus sequences as shown for DBL6ε. Each of these consensus sequences is specific for a given VB and DBL position in the VAR2CSA protein. By aligning and comparing DBL domains from the same position, we were able to determine all the variable blocks. These VB differ in number and length, depending of the position of the DBL in VAR2CSA but the significance of the length variation is unclear. The presence or absence of variability may provide an indication of exposed and non-exposed sequences, and may help to determine which DBL is the most solvent-exposed.

The most frequent variation mechanism is not substitutions or deletions of random amino acids but replacements of consensus sequences by others via recombination events [26], [31], [39], [40], [41]. A consensus sequence is VB specific and rarely found neither in another block nor DBL domain from another VAR2CSA, although selected homology blocks from DBL6ε (DBLpam6) are found within DBLγ, DBLδ and DBLε [10]. Nevertheless, one exception has been observed. The most likely mechanism for allelic diversity at antigenic loci is crossover recombination [42], [43] of *var2csa* with other *var* genes or pseudo genes. In the 3D7 genome, we did not find any consensus sequences identical to those of its *var2csa*, but we found a partial sequence of *var2csa* identical to another PfEMP1 of the 2155-3 genome (accession number: CQ848092). The *var* gene PFF1580c (accession number: CAG25137.7) also referred to as MAL6P1.6 (Figure S7) is expressed by parasites involved in infections targeting children [44] and is associated with severe disease [45]. Its membrane-proximal domain, DBL7ε, has VB3, VB4 and VB5 homologous to consensus sequences VB3-1, VB4-1 and VB5-4. In addition, it has five constant blocks (amino acids 39–51; 97–116; 140–156; 179– 191 and 233–241) of which four separating the three homologous VB are identical to those of the VAR2CSA domain DBL6ε of the 2155-3 strain. Moreover, this DBL7ε is able to bind CSA [46] and the gene is found to recombine [47]. Thus, these consensus sequences are not restricted to PAM parasites, which may explain why IgG of some never-pregnant individuals can react against VAR2CSA [17], [48], [49], [50]. This observation opens the possibility to explain why numerous pregnant women have antibodies against VB1 and VB5 although they have not been infected with *P. falciparum* during their pregnancy, and why 86% of studied infected women plasma recognised at least one of the 15 peptides, hypothesising that some of these characterized consensus sequences can be found within other *var* genes. In addition, it is likely the several women that we classified as "non-infected", indeed presented submicroscopic infections during their pregnancy [51] that boosted antibodies against a plasmodial strain.

Some studies conducted on peptides showed that a neutralizing monoclonal antibody against anthrax protective antigen recognise a linear epitope [52], a protective antibody against *Plasmodium chabaudi* recognise a linear epitope [53], and a peptide can be recognised by a protective antibody against Ebola virus [54]. The linear epitope carried by a synthetic peptide can induce antibodies that will protect mice against Semliki forest virus [55]. Some peptides have already been shown to be good immunogens [56], inducing strong T cell response. All these studies show that there is a real interest to design peptides as vaccine candidates. Thus, it is clear that linear epitopes can be the targets of protective antibodies, and that peptides can induce protective antibodies production. The principal aim of our work has been to define the best peptides for vaccine design. Here, we determined the most relevant B epitopes with regard to immunological and physico-chemical characteristics. The next step is testing the functionality of the induced antibodies.

Finally, we identified DBL6ε sequences that were the best and the least dominant epitope sequences located within each VB. Depending of its VB sequences, a given DBL6ε may constitute a good vaccine candidate or not. In a general manner, no particular DBL domain should be more immunogenic than another but the choice of the VB sequences must be more discriminate.

## Supporting Information

Figure S1 **Alignment of the DBL1X domain from VAR2CSA.** DBL1X domain sequences were aligned to determine the constant and variable blocks. Cysteins were numbered in bold, VB were highlighted in gray. Identical and homologous amino acids were coloured in red and blue, respectively.
(TIF)

Figure S2 **Alignment of the DBL2X domain from VAR2CSA.** DBL2X domain sequences were aligned to determine the constant and variable blocks. Cysteins were numbered in bold, VB were highlighted in gray. Identical and homologous amino acids were coloured in red and blue, respectively.
(TIF)

Figure S3 **Alignment of the CIDRpam domain from VAR2CSA.** CIDRpam domain sequences were aligned to determine the constant and variable blocks. Cysteins were numbered in bold, VB were highlighted in gray. Identical and homologous amino acids were coloured in red and blue, respectively.
(TIF)

Figure S4 **Alignment of the DBL3X domain from VAR2CSA.** DBL3X domain sequences were aligned to determine the constant and variable blocks. Cysteins were numbered in bold, VB were highlighted in gray. Identical and homologous amino acids were coloured in red and blue, respectively.
(TIF)

Figure S5 **Alignment of the DBL4ε domain from VAR2CSA.** DBL4ε domain sequences were aligned to determine the constant and variable blocks. Cysteins were numbered in bold, VB were highlighted in gray. Identical and homologous amino acids were coloured in red and blue, respectively.
(TIF)

Figure S6 **Alignment of the DBL5ε domain from VAR2CSA.** DBL5ε domain sequences were aligned to determine the constant and variable blocks. Cysteins were numbered in bold, VB were highlighted in gray. Identical and homologous amino acids were coloured in red and blue, respectively.
(TIF)

Figure S7 **The DBL6ε domain from the 2155**-**3 strain and the DBL7ε from the PFF1580c PfEMP1 were aligned.** Amino acids were coloured as in figure 5. VB were highlight in purple.
(TIF)
