# Supplementary figures and images for: Plasmodium falciparum Variability and Immune Evasion Proceed from Antigenicity of Consensus Sequences from DBL6ε; Generalization to All DBL from VAR2CSA
Source: PLoS One. 2013 Jan 25;8(1):e54882. doi: 10.1371/journal.pone.0054882 (PMC3555990; doi:10.1371/journal.pone.0054882)

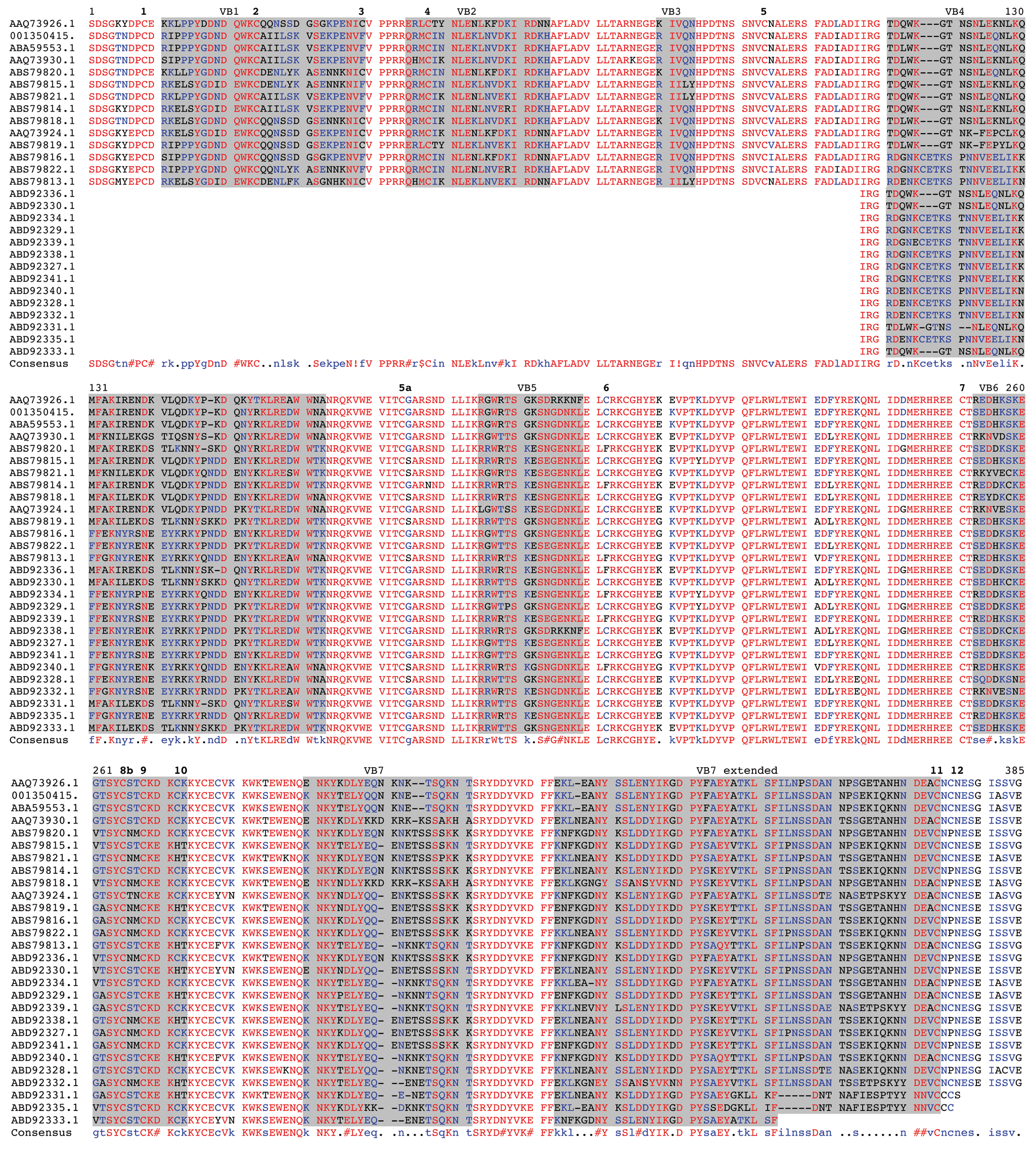

Supplement: Figure S1 — Alignment of the DBL1X domain from VAR2CSA. DBL1X domain sequences were aligned to determine the constant and variable blocks. Cysteins were numbered in bold, VB were highlighted in gray. Identical and homologous amino acids were coloured in red and blue, respectively. (TIF) [file pone.0054882.s001.tif]

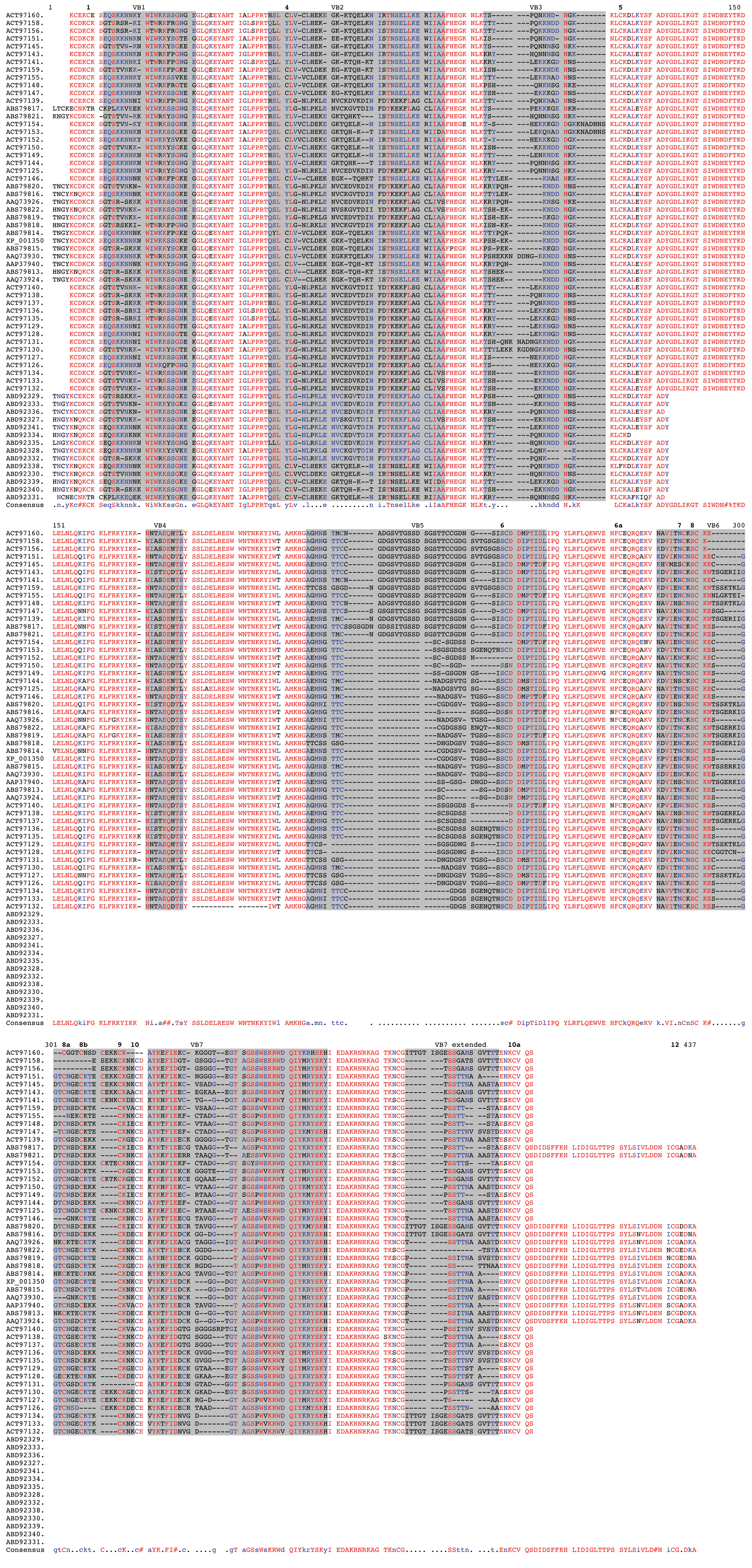

Supplement: Figure S2 — Alignment of the DBL2X domain from VAR2CSA. DBL2X domain sequences were aligned to determine the constant and variable blocks. Cysteins were numbered in bold, VB were highlighted in gray. Identical and homologous amino acids were coloured in red and blue, respectively. (TIF) [file pone.0054882.s002.tif]

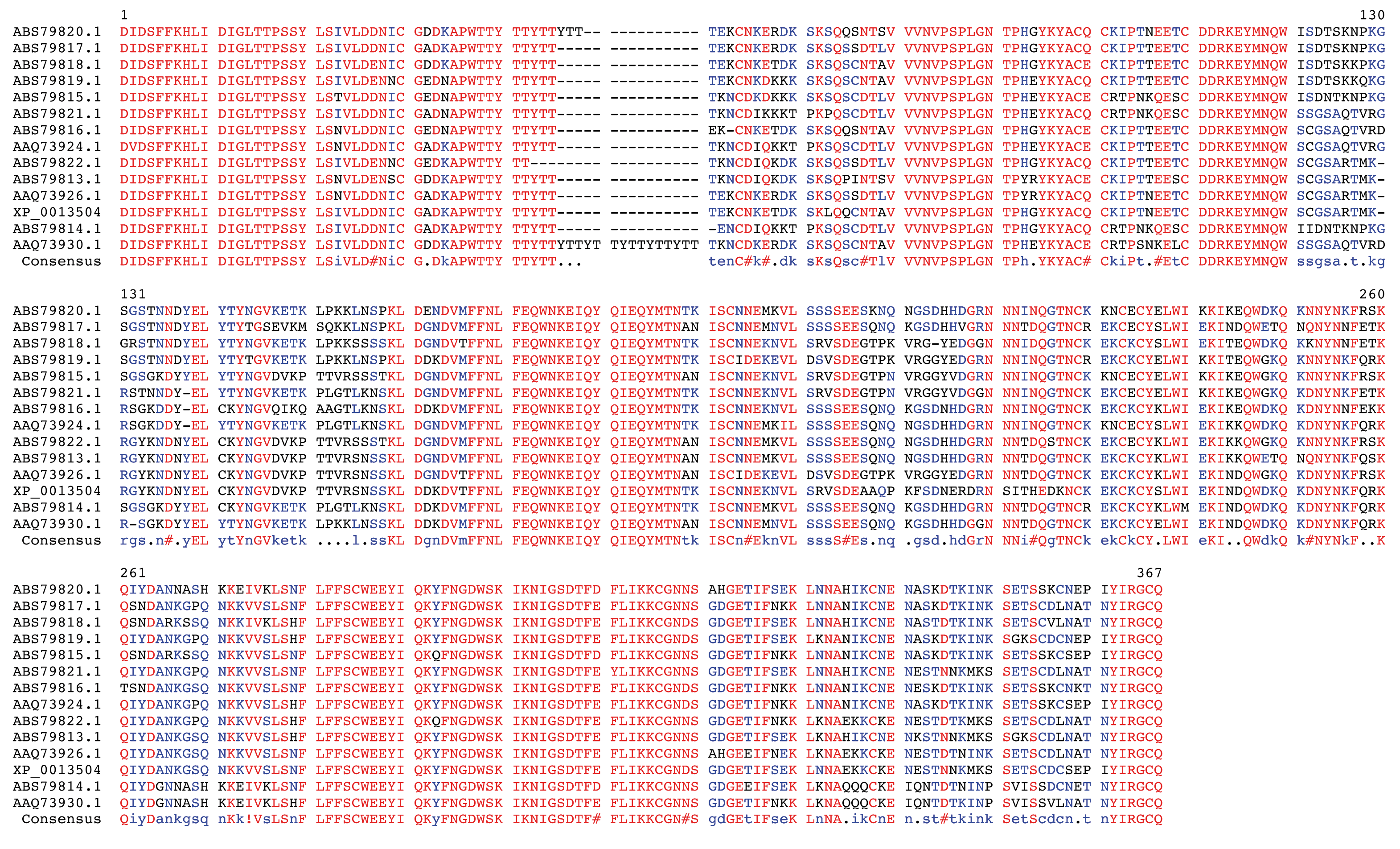

Supplement: Figure S3 — Alignment of the CIDRpam domain from VAR2CSA. CIDRpam domain sequences were aligned to determine the constant and variable blocks. Cysteins were numbered in bold, VB were highlighted in gray. Identical and homologous amino acids were coloured in red and blue, respectively. (TIF) [file pone.0054882.s003.tif]

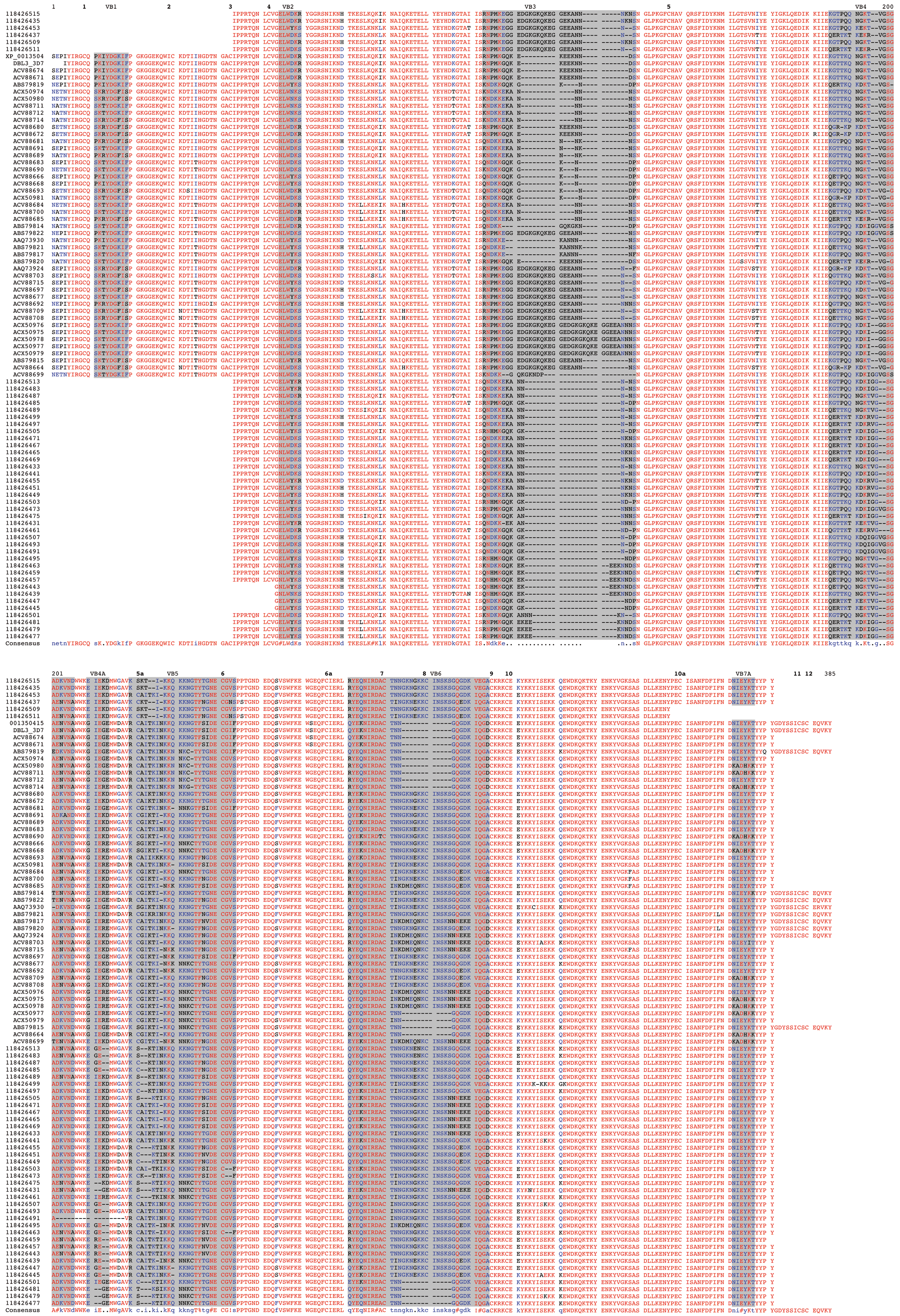

Supplement: Figure S4 — Alignment of the DBL3X domain from VAR2CSA. DBL3X domain sequences were aligned to determine the constant and variable blocks. Cysteins were numbered in bold, VB were highlighted in gray. Identical and homologous amino acids were coloured in red and blue, respectively. (TIF) [file pone.0054882.s004.tif]

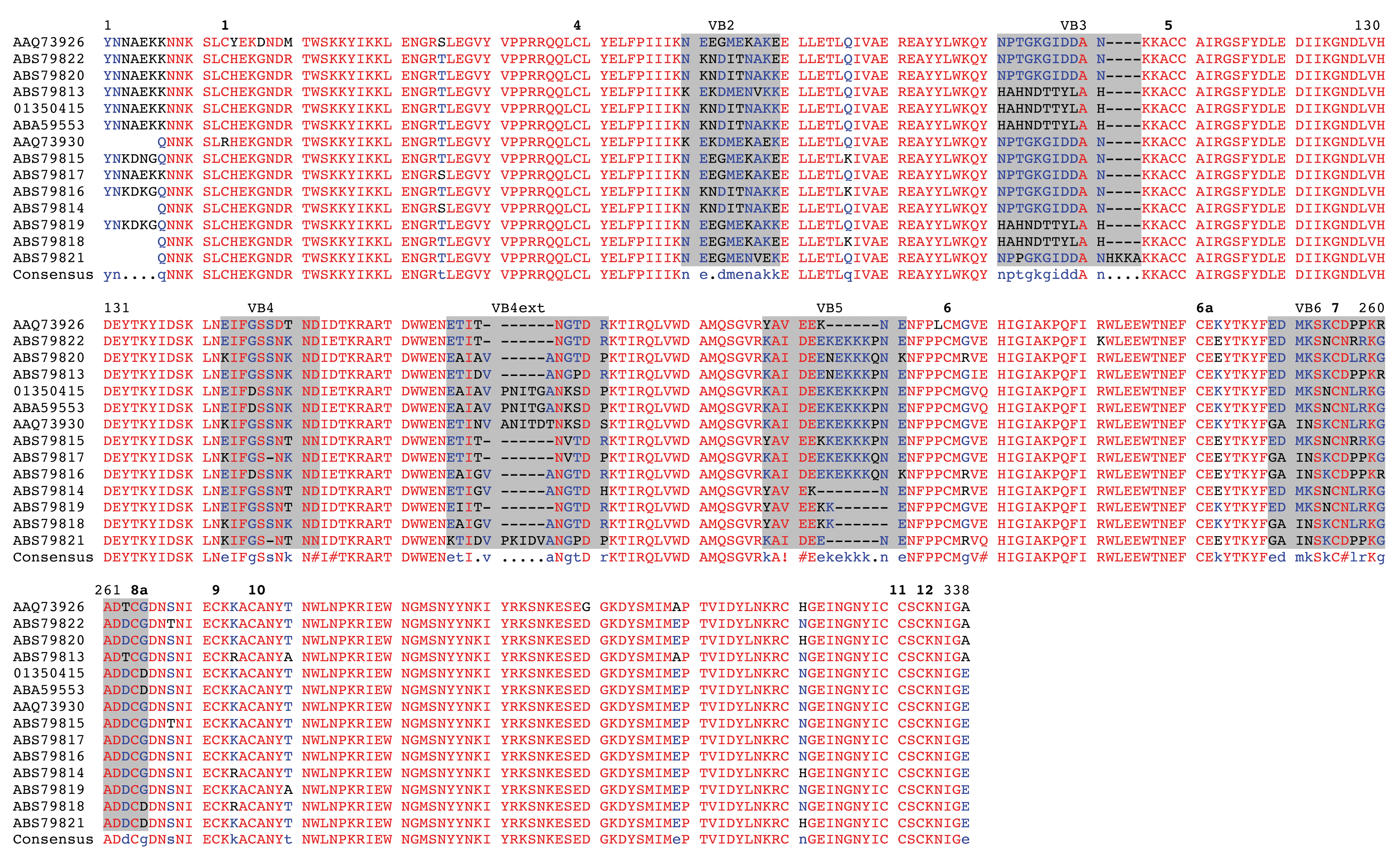

Supplement: Figure S5 — Alignment of the DBL4ε domain from VAR2CSA. DBL4ε domain sequences were aligned to determine the constant and variable blocks. Cysteins were numbered in bold, VB were highlighted in gray. Identical and homologous amino acids were coloured in red and blue, respectively. (TIF) [file pone.0054882.s005.tif]

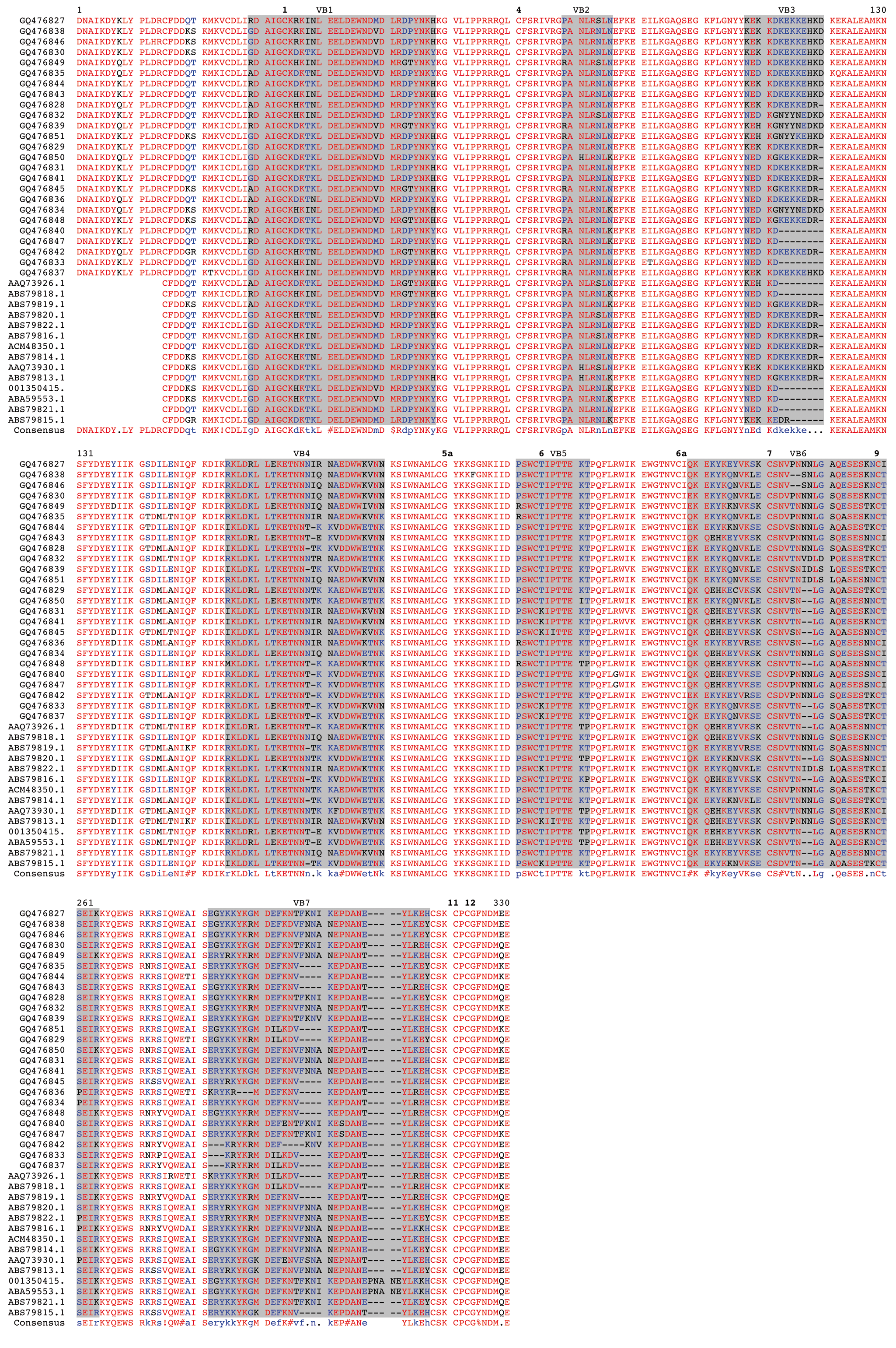

Supplement: Figure S6 — Alignment of the DBL5ε domain from VAR2CSA. DBL5ε domain sequences were aligned to determine the constant and variable blocks. Cysteins were numbered in bold, VB were highlighted in gray. Identical and homologous amino acids were coloured in red and blue, respectively. (TIF) [file pone.0054882.s006.tif]

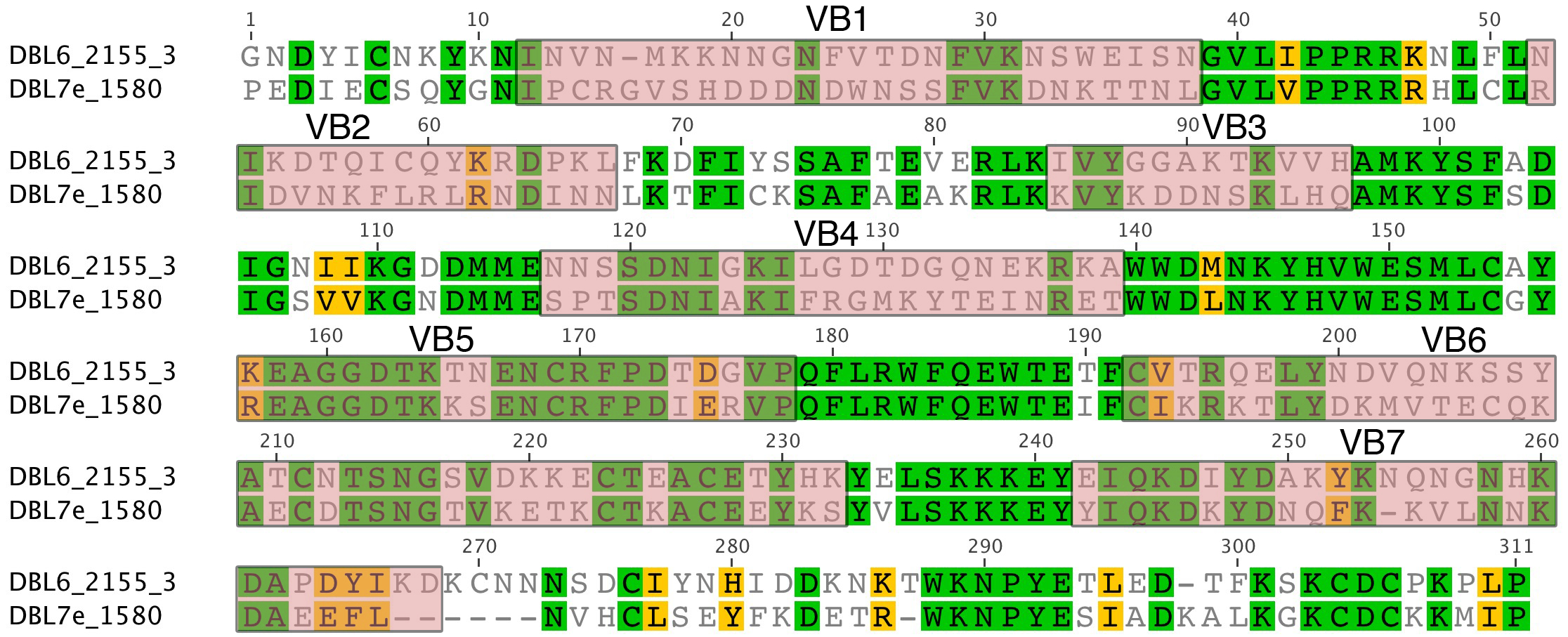

Supplement: Figure S7 — The DBL6ε domain from the 2155-3 strain and the DBL7ε from the PFF1580c PfEMP1 were aligned. Amino acids were coloured as in figure 5. VB were highlight in purple. (TIF) [file pone.0054882.s007.tif]
